# Supplementary material for: Grass supplementation to a pellet-based diet fails to enrich gut microbiomes with wild-like functions in captive-bred hares
Source: Microbiol Spectr. 2026 Mar 5;14(4):e03691-25. doi: 10.1128/spectrum.03691-25 (PMC13055366; doi:10.1128/spectrum.03691-25)
Supplement: Supplemental table and figures — Table S1 and Figures S1 to S5. [file spectrum.03691-25-s0001.docx]

# Supplementary data **Grass supplementation to a pellet-based diet fails to enrich gut microbiomes with wild-like functions in captive-bred hares**

**Table S1.** Phyla present in wild and captive hares and their differential abundance analysis.

| **Phylum** | **Total** | **Wild** | **Captive** | **Log-fold change** | **p-value** |
| --- | --- | --- | --- | --- | --- |
| **Bacteroidota** | 25.787±10.113 | 26.889±11.805 | 24.986±10.083 | 1.198 | 0.011 |
| **Spirochaetota** | 3.911±10.011 | 11.731±14.792 | 0.001±0.001 | 8.728 | 4.777e-07 |
| **Verrucomicrobiota** | 2.264±5.722 | 1.472±0.715 | 1.449±1.295 | 2.163 | 5.026e-03 |
| **Synergistota** | 0.96±0.852 | 1.865±0.83 | 0.45±0.39 | 3.356 | 8.143e-06 |
| **Bacillota_C** | 0.844±0.795 | 1.696±0.674 | 0.386±0.36 | 3.080 | 3.586e-05 |
| **Desulfobacterota** | 0.586±0.396 | 0.991±0.315 | 0.41±0.256 | 2.324 | 5.530e-05 |
| **Bacillota B** | 0.104±0.145 | 0.228±0.203 | 0.042±0.024 | 2.767 | 7.923e-07 |
| **Bacillota_A** | 57.243±12.618 | 48.636±13.52 | 59.846±10.725 | 0.918 | 0.918 |
| **Bacillota** | 4.118±7.202 | 5.066±11.152 | 4.949±5.297 | -1.454 | 0.191 |
| **Pseudomonadota** | 1.626±3.328 | 0.511±0.397 | 3.082±5.382 | 0.259 | 0.682 |
| **Patescibacteria** | 0.987±1.542 | 0.212±0.253 | 2.177±2.223 | -0.780 | 0.142 |
| **Actinomycetota** | 0.78±0.985 | 0.427±0.459 | 1.217±1.147 | 0.478 | 0.3383 |
| **Cyanobacteriota** | 0.708±2.081 | 0.277±0.614 | 0.916±3.024 | 0.260 | 0.800 |
| **Campylobacterota** | 1.08e-04±2.35e-04 | 7.1e-05±8.1e-05 | 1.27e-04±2.26e-04 | 1.397 | 0.1717 |


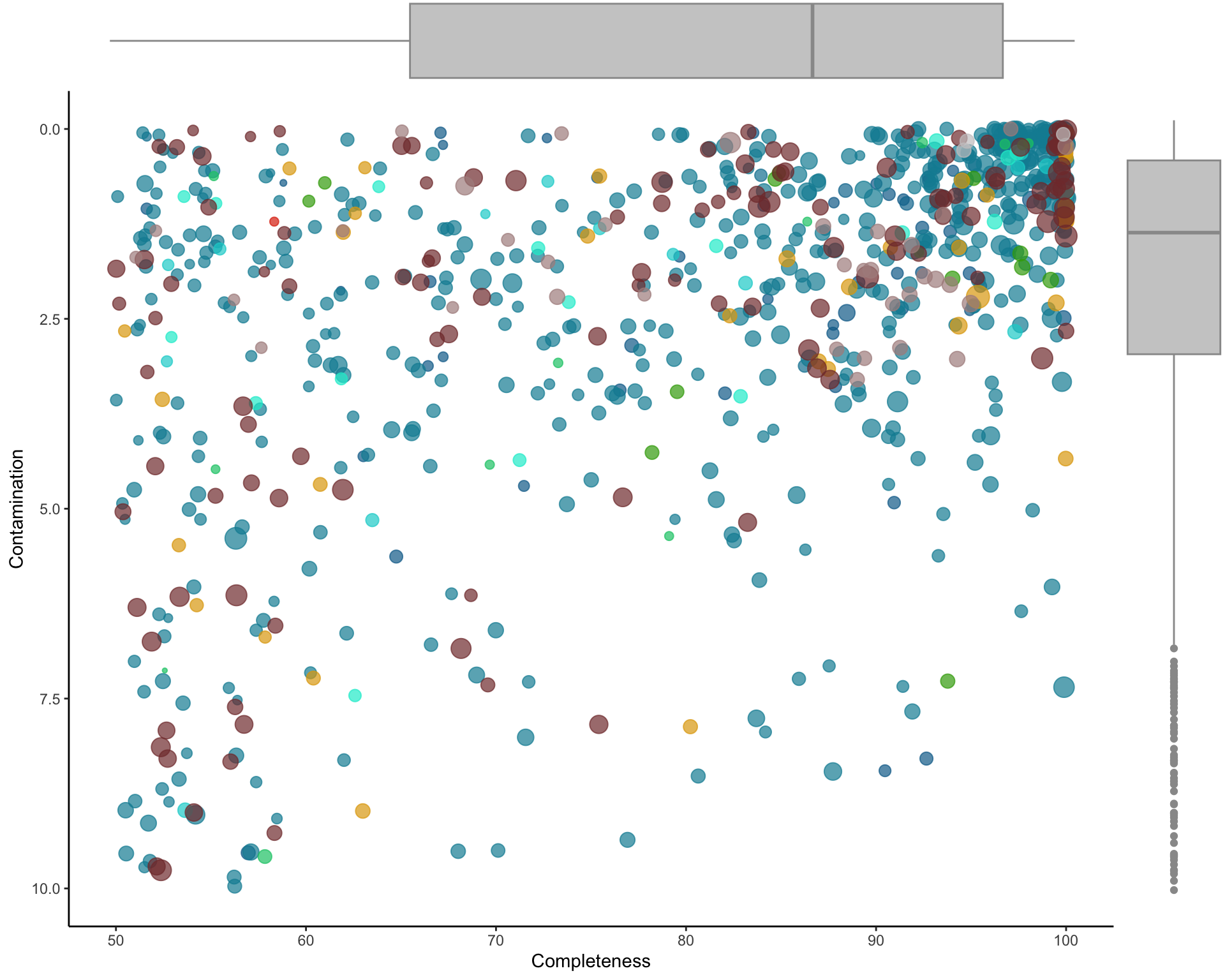


**Figure S1.** Biplot chart where MAGs are ordinated according to their completeness and contamination scores, and dot sizes indicate their genome size.


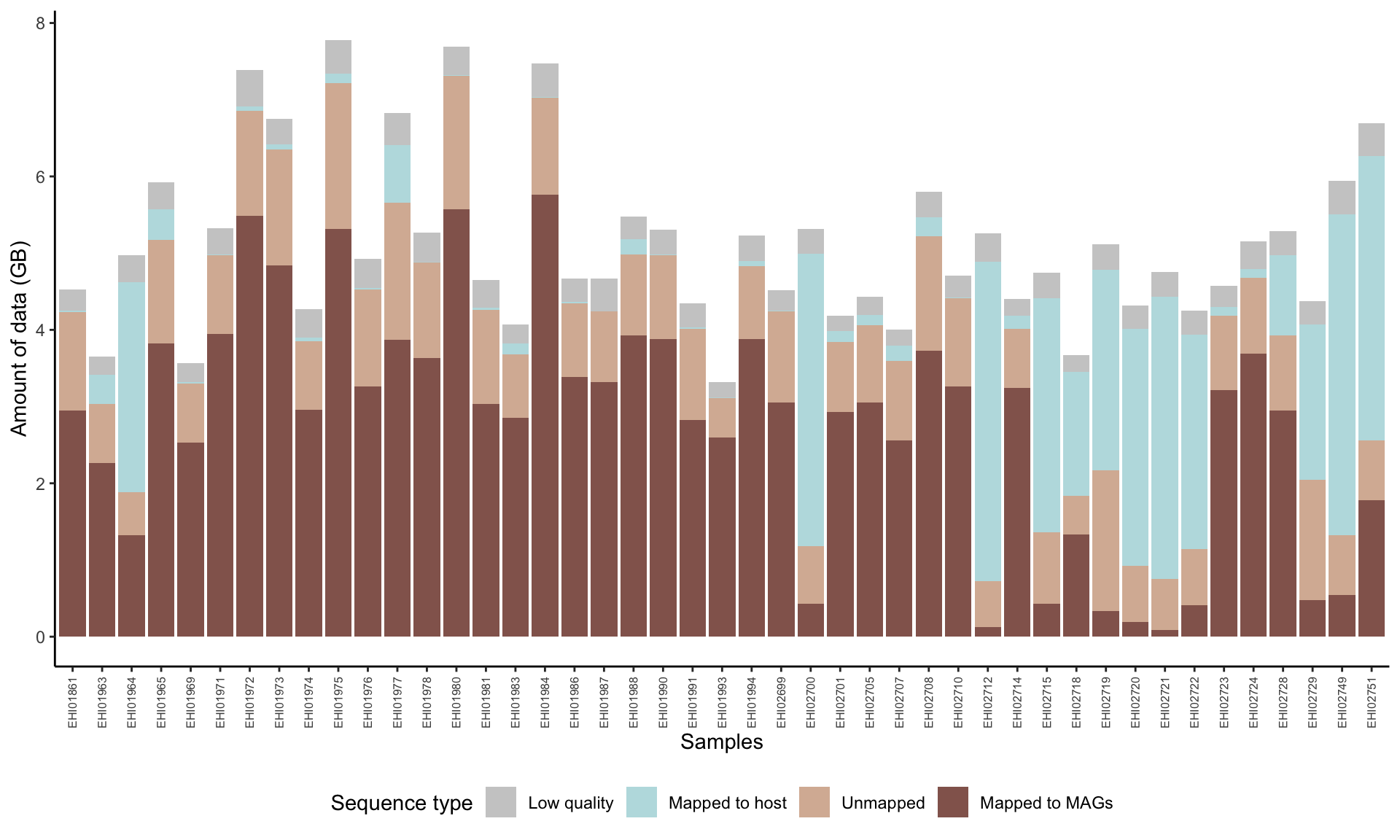


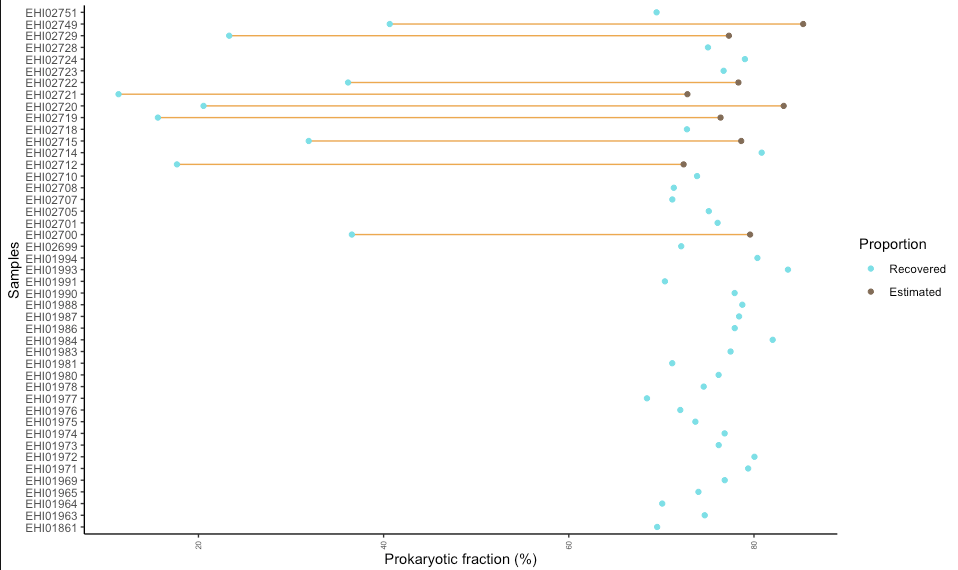


**Figure S2.** Amount of data generated for each sample and differences between estimated and captured microbial fractions. Nine samples (EHI02721, EHI02712, EHI02700, EHI02720, EHI02749, EHI02719, EHI02729, EHI02715, EHI02722) were removed from the analysis because the high amount of reads mapped to the host.

**
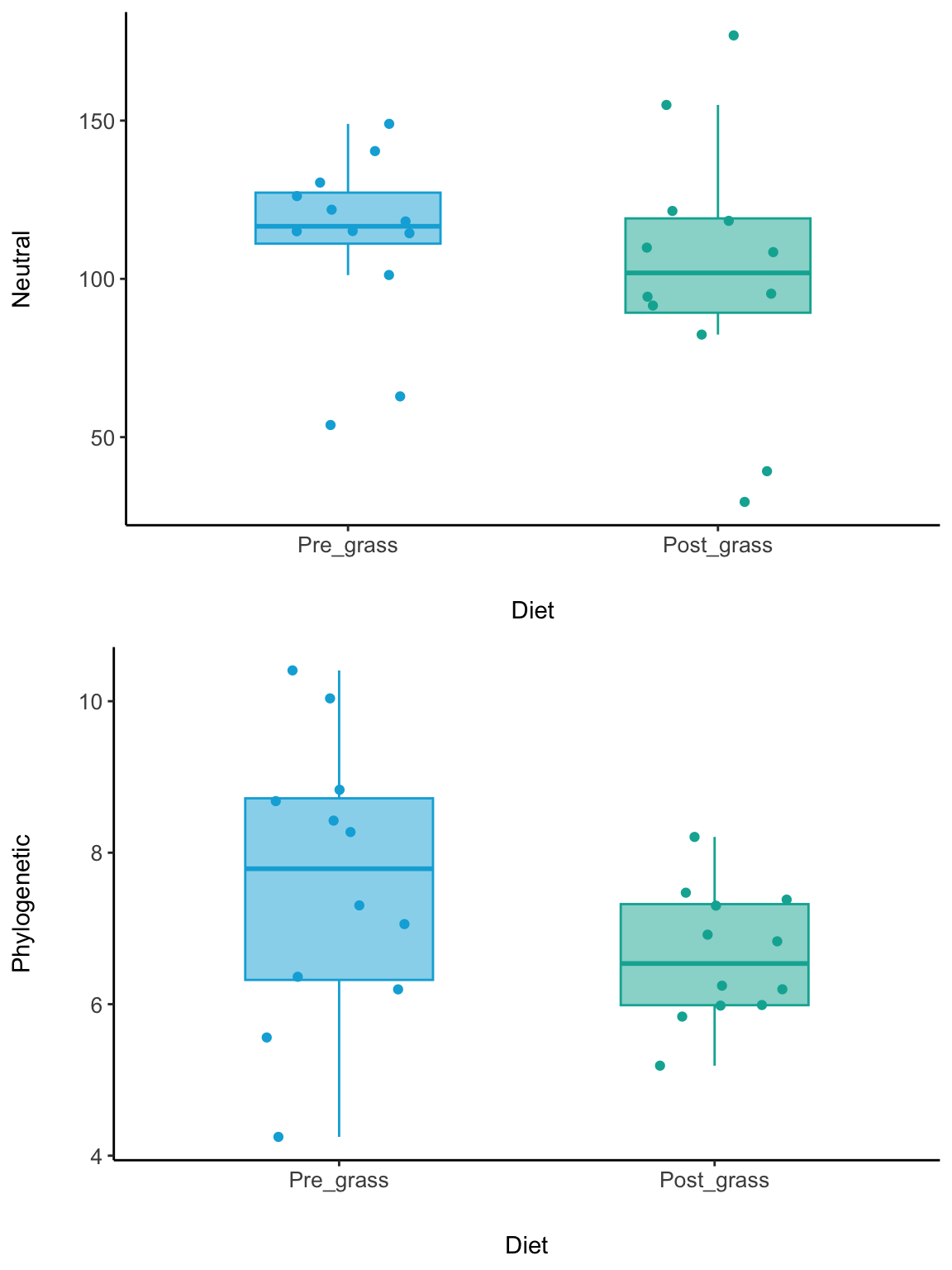
**

**Figure S3.** Neutral alpha diversity differences between individuals before and after grass was included in the diet.

**
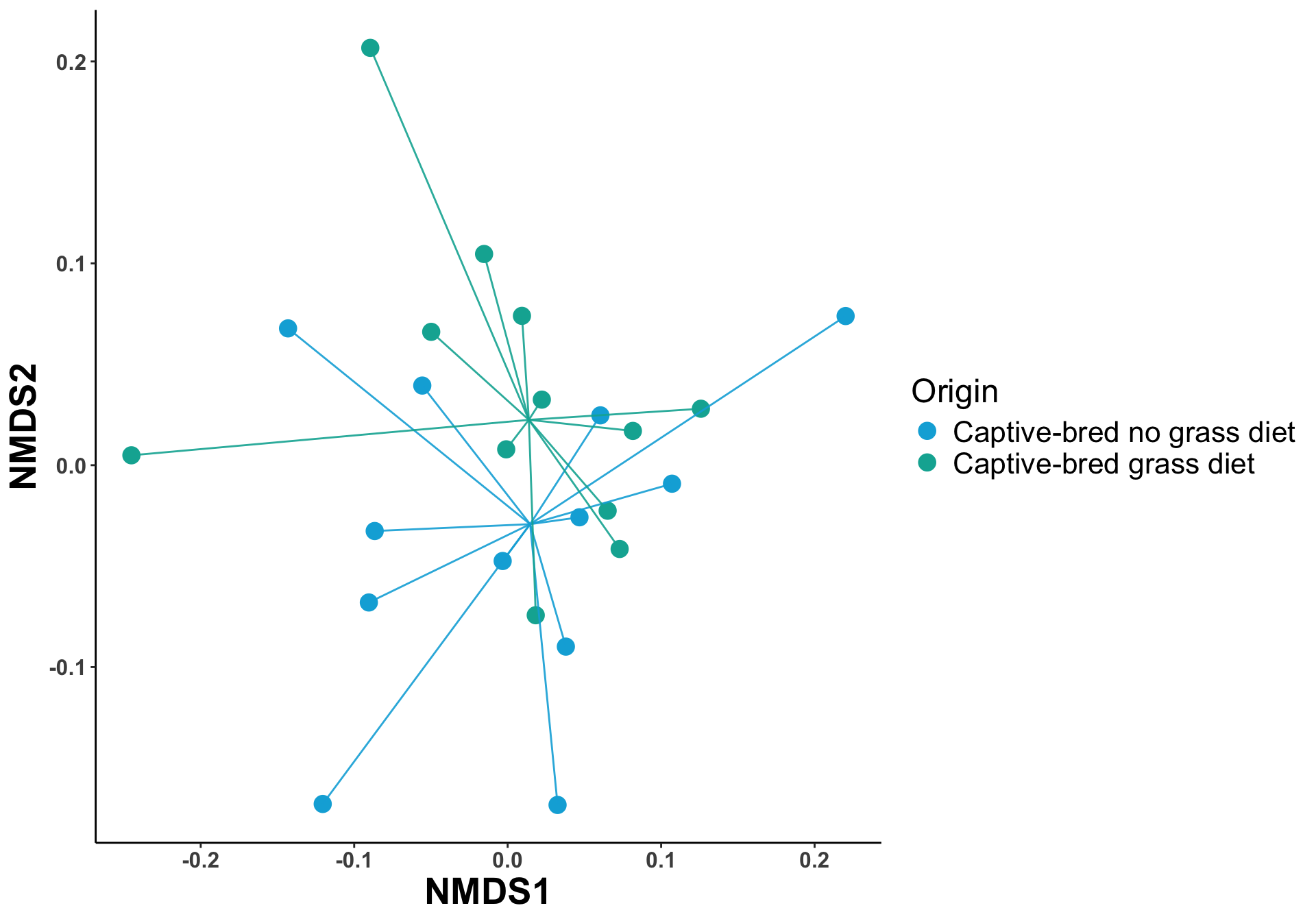
**

**Figure S4.**  NMDS ordination plot derived from pairwise dissimilarity values between samples based on Jaccard-type turnover for neutral beta diversity


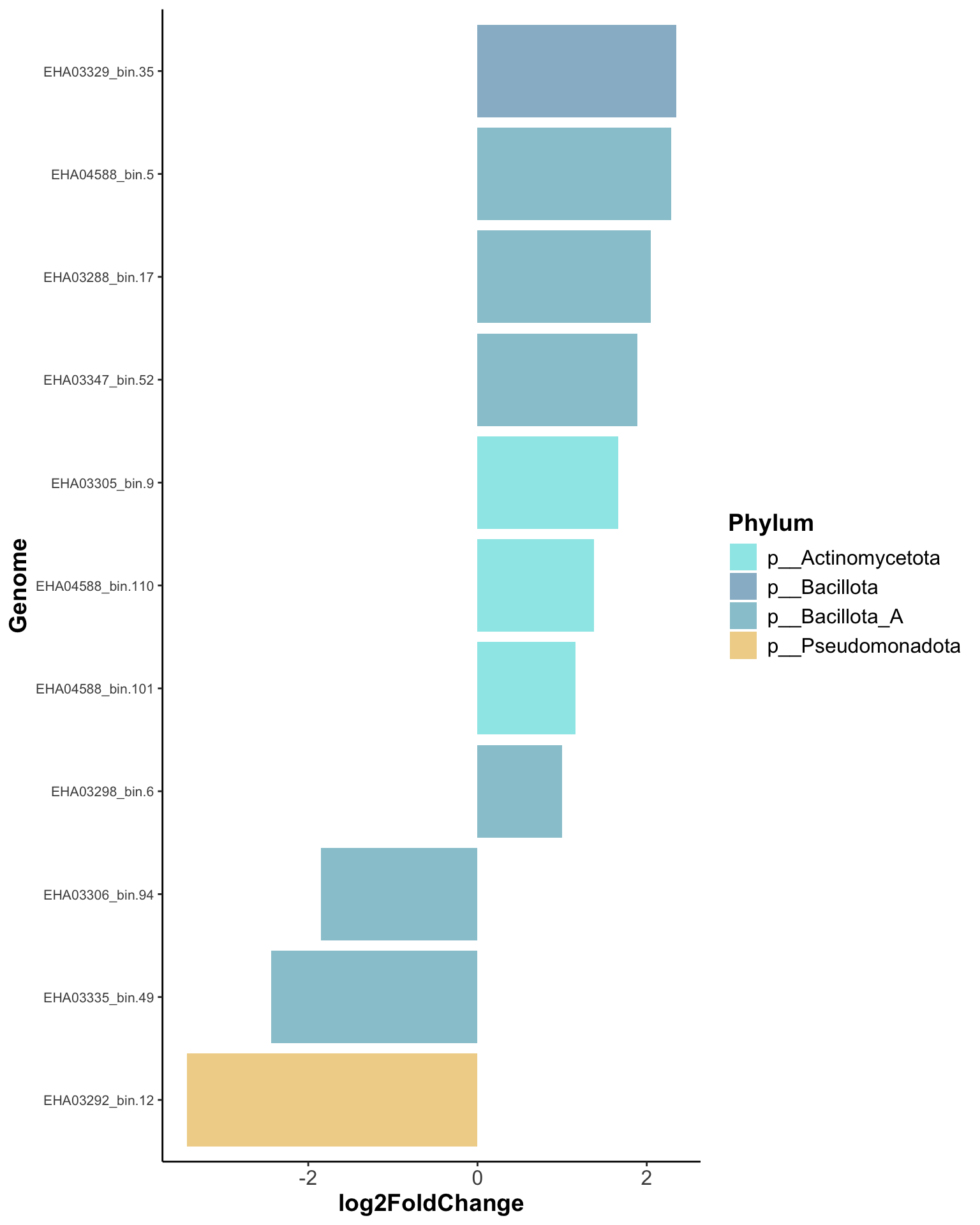


**Figure S5.** Differential abundance analysis between captive hares before and after grass was included in the diet.
